# Cross-Database Analysis Reveals Sensitive Biomarkers for Combined Therapy for ERBB2+ Gastric Cancer

**DOI:** 10.3389/fphar.2018.00861

**Published:** 2018-08-03

**Authors:** Zhen Xiang, Xia Huang, Jiexuan Wang, Jun Zhang, Jun Ji, Ranlin Yan, Zhenggang Zhu, Wei Cai, Yingyan Yu

**Affiliations:** ^1^Department of Surgery, Ruijin Hospital, Shanghai Institute of Digestive Surgery, Shanghai Key Laboratory of Gastric Neoplasms, Shanghai, China; ^2^Department of Disease Prevention and Control, Ruijin Hospital, Shanghai Jiao Tong University School of Medicine, Shanghai, China

**Keywords:** gastric cancer, HER2, RARA, lapatinib, ATRA

## Abstract

Exploring ERBB2-related pathways will help us finding sensitive molecules and potential combined therapeutic targets of ERBB2-targeted therapy for ERBB2+ gastric cancer (GC). In this study, we performed a cross-databases study focused on ERBB2+ GC. The data of ERBB2+ GC deposited in the cancer genome atlas (TCGA), gene expression omnibus (GEO), InBio Map^TM^, cancer cell line encyclopedia (CCLE), and cancer therapeutics response portal (CTRP) were analyzed. The correlation of expression levels of candidate and IC50 of candidate genes-targeted drugs were verified on NCI-N87 and MKN-45 GC cell lines. We found that RARA, THRA, CACNB1, and TOP2A are drug sensitive biomarkers of ERBB2-targeted treatment with FDA-approved drugs. All these genes act through Myc signaling pathway. Myc is the downstream hub gene of both ERBB2 and RARA. The expression of RARA, THRA, and CACNB1 were negatively correlated with Myc activation, while ERBB2 and TOP2A positively correlated with Myc activation. SH3BGRL3, SH3BGRL, and NRG2 were identified as potential ligands of ERBB2. The ERBB2+ GC with RARA amplification demonstrated better prognosis than those without RARA amplification, while overexpression of NRG2 and SH3BGRL correlated with poor prognosis in ERBB2+ GC. About 90% of ERBB2+ GC was compatible with chromosome instability (CIN) subtype of TCGA, which overlaps with intestinal-type GC in Lauren classification. In validating experiments, combination of Lapatinib and all-trans retinoic acid (ATRA) synergistically suppresses cell growth, and accompanied by decreased expression of MYC. In conclusions, we identified several predicting biomarkers for ERBB2-targeted therapy and corresponding histological features of ERBB2+ GC. Combination of ERBB2 antagonist or RARA agonist may be effective synergistic regimens for ERBB2+ GC.

## Introduction

Gastric cancer (GC) is one of the most common cancers and the third leading cause of mortality worldwide (Ferlay et al., 2015). In China, both of the morbidity and mortality of GC rank the second (Chen et al., 2016). The major therapeutic approaches for GC are surgery, adjuvant chemotherapy and targeted therapy. The targeted drugs approved by FDA are trastuzumab (for ERBB2), cetuximab (for EGFR), and ramucirumab (for VEGF2) (Van Cutsem et al., 2012; Wilke et al., 2014). Lapatinib, a small-molecule inhibitor of ERBB2, plus paclitaxel demonstrated activity in the second-line treatment of ERBB2+ GC (Hecht et al., 2016). In clinics, ERBB2+ GC is defined by scoring 3^+^ by immunohistochemistry or copy number amplification by FISH (He et al., 2013). In general, ERBB2+ GC accounts for about 13% of all GC cases (Bass et al., 2014). A phase III ToGA study showed that the incidence of ERBB2+ GC is up to 22% (Bang et al., 2010). Because the prognosis of ERBB2+ GC is poor, clarifying the mechanisms of drug sensitivity of ERBB2+ GC will be of clinical significance in ERBB2-targeted therapy (Wang et al., 2017). Currently, trastuzumab plus fluorouracil and platinum can effectively improve overall survival of ERBB2+ GC patients, but the response rate was only 32–68% (Ock et al., 2015). Finding additional therapeutic targets for combined therapy will benefit more ERBB2+ GC patients.

ERBB2 is located in chromosome 17q21.2, where some common oncogenes or tumor suppressor genes, such as TOP2A, TAU, p53, and HIC-1 are located (Zhang and Yu, 2011). Our previous studies confirmed that re-activation of tumor suppressor HIC-1 by small-activating RNAs inhibits cell division, growth and invasion (Zhao et al., 2014, 2015). Retinoic acid receptor alpha (RARA) is another gene located on chromosome 17q21.2. Paroni et al. (2012) reported that combination of RARA agonist and ERBB2-targeted drug demonstrated a synergistic anticancer activity in breast cancer. It suggested that some novel therapeutic targets for ERBB2+ GC may harbor on chromosome 17. In this study, we analyzed ERBB2-related pathways and explored potential drug sensitivity biomarkers that could be used as reference for targeted therapy of ERBB2+ GC.

## Materials and Methods

### Data Extraction and Data Mining

The data of gene expression, copy number variation, tissue images and clinical information of 413 GC cases was extracted from The Cancer Genome Atlas database (TCGA^1^) and cBioPortal database^2^ (Cerami et al., 2012; Gao et al., 2013; Bass et al., 2014). The data of gene expression and copy number variation of GC was also extracted from GSE62717 and GSE57302 in gene expression omnibus (GEO) database^3^ (Qian et al., 2014; Cristescu et al., 2015). The data of protein-protein interaction was used in InBio Map^TM^ database^4^ (Li et al., 2017). The information of Lapatinib IC50 of 17 GC cell lines were extracted from database of Cancer Cell Line Encyclopedia (CCLE^5^) (Barretina et al., 2012). The information of Afatinib IC50 of 16 GC cell lines was extracted from database of Cancer Therapeutics Response Portal (CTRP^6^) (Basu et al., 2013). Kyoto Encyclopedia of Genes and Genomes (KEGG) pathway database is used for explaining functions and biology of genes^7^. The analytic results were confirmed for proteins expression using The Human Protein Atlas^8^.

#### Gastric Cancer Cell Lines and Cell Culture

Human GC cell lines NCI-N87 and MKN45 were purchased from the type Culture Collection of Chinese Academy of Sciences (Shanghai, China). All cell lines were cultured in RPMI-1640 containing 10% fetal bovine serum (FBS) at 37°C in a humidified incubator with 95% air and 5% CO_2_.

#### Cell Viability Assay

Firstly, 5000/well NCI-N87 or MKN45 cells were placed in 96 well plates (100 μl/well). Cells were treated by different concentrations of Lapatinib (Selleck, Houston, TX, United States) and All-Trans Retinoic Acid (ATRA, Selleck, Houston, TX, United States), respectively. CCK-8(10 μl/well) was used and OD value was measured at 450 nm by spectrophotometry (BioTek, VT, United States) at different time points.

#### Western Blot

The cytoplasmic protein and nuclear protein were extracted, respectively by extraction kit (Cat. P0027, Beyotime, Shanghai, China) according to the protocols. Western blot was performed as previously described (Duan et al., 2016). The antibodies used in this study were as following: HRP-conjugated mouse monoclonal GAPDH (1: 5000, Cat. HRP-60004, Proteintech Group, Inc. Wuhan, China), rabbit monoclonal Histone H3 (1: 5000, Cat. ab1791, Abcam, Cambridge, United Kingdom), and rabbit monoclonal c-MYC (1:1000, Cat. ab32072, Abcam, Cambridge, United Kingdom). Of those, GAPDH and Histone H3 were used as internal controls for cytoplasmic protein and nuclear protein, respectively.

### Statistical Analysis

EdgeR package in R language was used to screen differentially expressed genes (Robinson et al., 2010). We plotted OncoPrinter map based on gene copy number data from TCGA and cBioPortal platforms. Pheatmap package in R language was utilized to plot heatmap. Receiver operating characteristic (ROC) was plotted and an optimal cutoff value was selected for biomarkers from clinical samples. The median value was used as cutoff value for cancer cell lines. Survival rate was calculated by Kaplan-Meier method and log-rank test. GSEA software was applied to perform gene set enrichment analysis (Subramanian et al., 2005). Pearson correlation between RNA levels and IC50 was analyzed by Pearson test. CompuSyn software (ComboSyn Inc., Paramus, NJ, United States) was used to calculating combination index (CI) of drugs synergistic effects. CI < 1, =1, and >1 indicate synergistic, additive and antagonistic effects, respectively (Yu et al., 2018). Other statistical analysis was performed by using GraphPad Prism 6.0 (Inc., La Jolla, CA, United States). *P*-value less 0.05 was considered statistically significant.

## Results

### ERBB2+ GC Showed Amplification of RARA, THRA, CACNB1, and TOP2A

**FIGURE 1:**
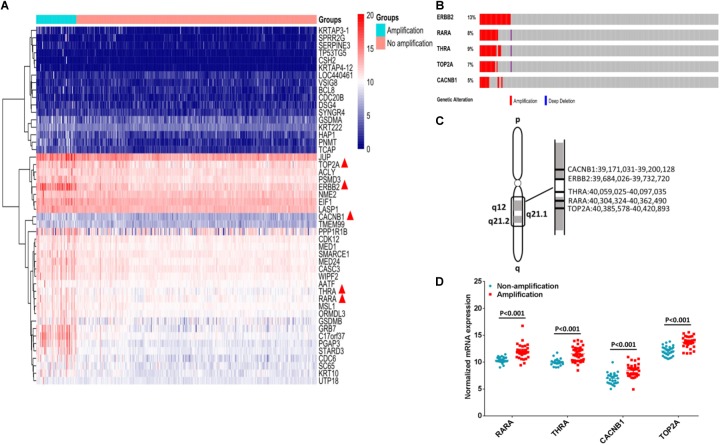
Identification of potential targets for combination therapy of ERBB2+ GC. **(A)** Forty-seven genes were up-regulated in ERBB2+ GC (*n* = 59 vs. *n* = 354, *P* < 0.0001). Among them, five genes are molecular targets of FDA approved drugs (indicated by red triangle). **(B)** The gene copy number amplification of ERBB2, RARA, THRA, TOP2A, and CACNB1. **(C)** The five genes ERBB2, RARA, THRA, TOP2A, and CACNB1 located in adjacent region of chromosomal 17q. **(D)**. The mRNA expression of RARA, THRA, CACNB1, and TOP2A in ERBB2+ GC and ERBB2– GC (*P* < 0.001).

Among 413 cases of GC from TCGA dataset, ERBB2+ GC accounted for 14.29% (59/413 cases), and the others were ERBB2- GC (354/413 cases). We compared the differential expressed genes and copy number variations between ERBB2+ and ERBB2- GC groups. Forty-seven differential expressed genes were selected based on the likelihood ratio (LR) and *P*-value (LR > 100, *P* < 0.0001; **Figure 1A**). Four out of these 47 genes are the molecular targets of FDA-approved drugs. They are CACNB1 (calcium voltage-gated channel auxiliary subunit beta 1) for amlodipine, TOP2A (DNA topoisomerase II alpha) for etoposide, RARA for ATRA and THRA (thyroid hormone receptor, alpha) for tiratricol. These genes were selected as potential targets of combination therapy with ERBB2-targeted drugs.

Gene amplification of RARA, THRA, CACNB1, and TOP2A was often accompanied by ERBB2 amplification (**Figure 1B**). These genes were located in adjacent region of chromosomal 17q (**Figure 1C**). In ERBB2+ GC, the rate of gene amplification was 57.63% (34 out of 59) for RARA, 62.71% (37 out of 59 (62.71%) for THRA, 50.85% (30 out of 59) for TOP2A, and 54.24% (32 out of 59) for CACNB1. The elevated mRNAs were also identified for cases with gene amplification, compared to cases without gene amplification (**Figure 1D**, *P* < 0.001).

### Amplified Genes of Chromosome 17q Were Myc Pathway-Related Genes

**FIGURE 2:**
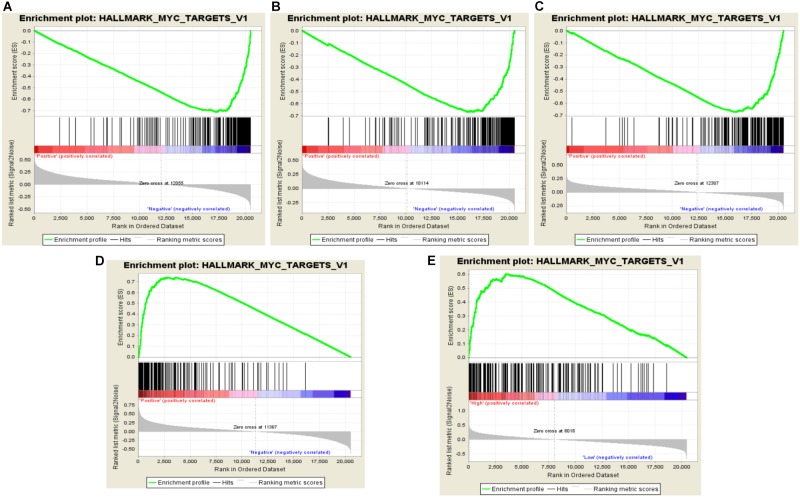
Identification of RARA, THRA, CACNB1, and TOP2A related pathways in ERBB2+ GC. **(A–C)** RARA, THRA, and CACNB1 are negatively related to Myc pathway activation with minus ES value. **(D,E)** TOP2A and ERBB2 are positively related to Myc pathway activation with plus ES value. The green curve represents enrichment score. The highest point is the enrichment score value in Myc pathway. The ES value indicates the correlation between gene and Myc pathway. The black bar codes represent genes in pathway, which are ordered according to their expression levels. The left end or right end genes are leading edge subset strongly contributed to ES value. The bottom numbers represent order of expression levels from high to low in genome.

Gene set enrichment analysis (GSEA) is a visualized method of the gene ontology analysis, which could help researchers finding a group of functional genes in a signaling pathway. Through GSEA, we found that all amplified genes of 17q are Myc-related genes. Of those, RARA, THRA, and CACNB1 were negatively correlated with Myc activation, which revealed minus value of enrichment score (ES). For instance, the ES value of RARA is −1.93 (FDR = 0.017, *P* = 0.004, **Figure 2A**), THRA is −1.85 (FDR = 0.035, *P* = 0.010, **Figure 2B**), and CACNB1 is −1.89 (FDR = 0.028, *P* = 0.004, **Figure 2C**). TOP2A and ERBB2 were positively associated with Myc activation (ES = 2.05, FDR = 0.002, *P* < 0.001, **Figure 2D** and (ES = 1.88, FDR = 0.077, *P* = 0.018, **Figure 2E**). These results implied that agonists of RARA, THRA, and CACNB1 or inhibitors of TOP2A or ERBB2 might be combined together for the treatment of ERBB2+ GC.

### The Association Between Amplified Genes and Biological Significance

**FIGURE 3:**
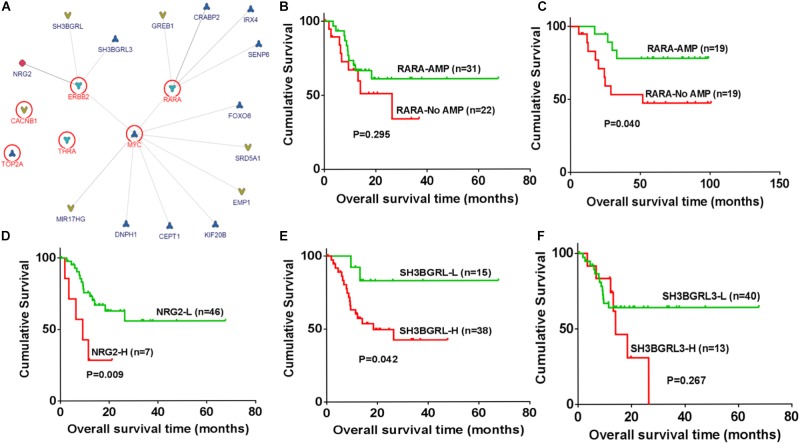
Identification of regulatory association for amplified genes and biological significances. **(A)** SH3BGRL3, SH3BGRL, and NGR2 are identified as potential ligands of ERBB2, while Myc is the downstream hub of ERBB2 and RARA based on InBio Map^TM^ analysis. **(B)** In ERBB2+ GC, RARA amplification shown better prognosis in TCGA cohort (HR = 0.628, 95% CI 0.242–1.539; *P* = 0.295). **(C)** In ERBB2+ GC of GEO datasets, RARA amplification shown better prognosis than that without RARA amplification (HR = 0.312, 95% CI 0.104–0.942; *P* = 0.040). **(D)** In ERBB2+ GC of TCGA cohort, overexpression of NRG2 was significantly correlated with poor prognosis (NRG2-L vs. NRG2-H, HR = 0.286, 95% CI 0.265–0.592, *P* = 0.009). **(E)** High expression of SH3BGRL was significantly correlated with poor prognosis (SH3BGRL-L vs. SH3BGRL-H, HR = 0.248, 95% CI 0.144–0.960, *P* = 0.042). **(F)** No significance was found for SH3BGRL3 (SH3BGRL3-L vs. SH3BGRL3-H, HR = 0.600, 95% CI 0.202–1.55, *P* = 0.267).

The association and pathway of amplified genes were analyzed by InBio Map^TM^. SH3BGRL3, SH3BGRL, and Neuregulin 2 (NRG2) are three candidate ligands of ERBB2. Both RARA and ERBB2 are upstream genes in Myc pathway (**Figure 3A**). To clarify the clinical significance of amplified genes in ERBB2+ GC, these genes in two datasets from TCGA and GEO were further analyzed. The tendency of better prognosis was noted in RARA amplification group, compared to non-RARA amplification group of ERBB2+ GC in both TCGA cohort (HR = 0.628, 95% CI 0.242–1.539, *P* = 0.295, **Figure 3B**), and GEO cohort (HR = 0.312, 95% CI 0.104–0.942, *P* = 0.04, **Figure 3C**). In ERBB2+ GC of TCGA cohort, high levels of NRG2 and SH3BGRL were significantly related to poor prognosis (*P* = 0.009 and *P* = 0.042, respectively, **Figures 3D,E**), but not for SH3BGRL3 (*P* = 0.267, **Figure 3F**). These results implied the ligand function of NRG2 and SH3BGRL in cancer progression of ERBB2+ GC.

### The Relationship Between Amplified Genes and Sensitivity to ERBB2-Targeted Drugs

**FIGURE 4:**
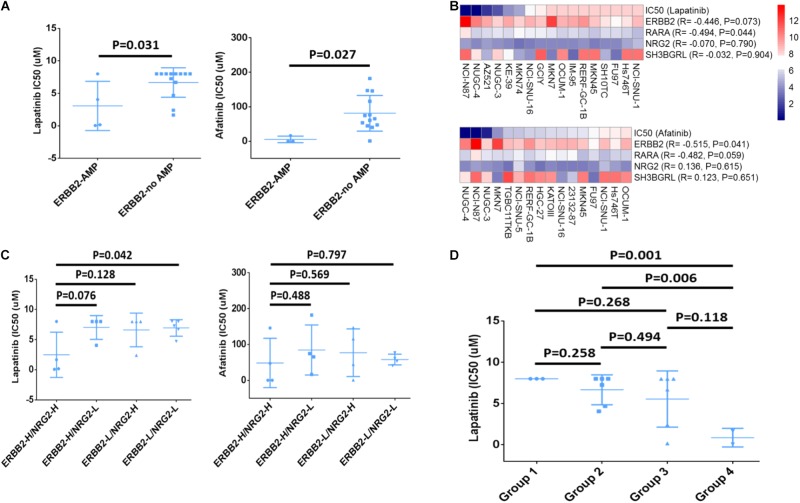
Drug sensitivity analysis of ERBB2, RARA, NRG2, and SH3BGRL. **(A)** GC cells with ERBB2 amplification showed lower IC50 (μM) to Lapatinib (*P* = 0.031) and Afatinib (*P* = 0.027) than those without ERBB2 amplification. **(B)** Relationship between IC50 of ERBB2-targeted drugs Lapatinib and Afatinib and mRNA expression of ERBB2, RARA, NRG2, and SH3BGRL. **(C)** IC50 of ERBB2-targeted drugs Lapatinib and Afatinib in cells with different mRNA expression, including ERBB2-H/NRG2-H, ERBB2-H/NRG2-L, ERBB2-L/NRG2-H, and ERBB2-L/NRG2-L group. **(D)** IC50 of Lapatinib on GC cells with different mRNA expression, including Group 1: NRG2-L/ERBB2-L/RARA-L (*n* = 3); Group 2: higher expression of any one out of NRG2, ERBB2, and RARA (*n* = 6); Group 3: lower expression of any one out of NRG2, ERBB2, and RARA (*n* = 6); and Group 4: NRG2-H/ERBB2-H/RARA-H (*n* = 2).

Since the gene copy number, mRNA level and IC50 value to ERBB2-targeted drugs of multiple cancer cell lines were deposited in CCLE database, the drug responsive status of 17 GC cell lines was analyzed. Among those, four cell lines (NCI-N87, KE39, NUGC-4, and MKN-7) were ERBB2-amplified cell lines that showed lower IC50 (μM) to drug Lapatinib than other GC cell lines (*P* = 0.031, **Figure 4A**, left). The ERBB2-amplified cell lines also showed lower IC50 (μM) to drug Afatinib than other cell lines (*P* = 0.027, **Figure 4A**, right). The CNV of ERBB2 or RARA and their responses to targeted drugs Lapatinib or Afatinib of GC cell lines were listed in **Table 1**. Cell line NCI-N87 were more sensitive to ERBB2-targeted drugs, which is related to dual amplification of ERBB2 and RARA, while MKN7 was not sensitive to ERBB2-targeted drugs, which is related to non-amplification of RARA.

**Table 1:** The correlation of CNV of ERBB2 or RARA with sensitivity to Lapatinib or Afatinib in ERBB2+ GC cells.

Cell lines	ERBB2 amplification	RARA amplification	Lapatinib IC50 (μM)	Afatinib IC50 (μM)
NCI-N87	Yes	Yes	0.066	0.141
NUGC-4	Yes	No	0.171	0.077
KE39	Yes	No	4.056	NA
MKN7	Yes	No	8.000	16.50

### The Relationship of Expression of ERBB2, RARA, NRG2, and SH3BGRL and Sensitivity to ERBB2-Targeted Drugs

ERBB2 mRNA overexpression was often significantly related to lower IC50 to Lapatinib (*R* = −0.446, *P* = 0.073) and Afatinib (*R* = −0.515, *P* = 0.041). Similarly, overexpression of RARA mRNA was significantly associated with lower IC50 to Lapatinib (*R* = −0.494, *P* = 0.044) and Afatinib (*R* = −0.482, *P* = 0.059). However, there was no significant correlation for NRG2 and SH3BGRL mRNA expression and drug sensitivity (**Figure 4B**). Nevertheless, GC cases with overexpression of ERBB2 and NRG2 (ERBB2-H/NRG2-H) were more sensitive to Lapatinib, compared to low gene expression of ERBB2-L/NRG2-L group (*P* = 0.042, **Figure 4C**, left). However, there was no significant difference between ERBB2-H/SH3BGRL-H group and ERBB2-L/NRG2-L group for drug Afatinib (**Figure 4C**, right).

The relationship of simultaneous overexpression of NRG2, ERBB2, and RARA with Lapatinib sensitivity was listed in **Table 2**. Group 1: NRG2-L/ERBB2-L/RARA-L; Group 2: high expression of any one of NRG2, ERBB2, or RARA; Group 3: lower expression of any one of NRG2, ERBB2, or RARA; and Group 4: NRG2-H/ERBB2-H/RARA-H. The IC50 to Lapatinib of Group 4 was the lowest (*P* = 0.001). Group 3 is better than that in Group 2 for sensitivity to Lapatinib (**Figure 4D**).

**Table 2:** The association of Lapatinib sensitivity, mRNA expression and gene amplification of ERBB2, RARA, and NRG2.

GC cell lines	ERBB2 mRNA	ERBB2 Amp	RARA mRNA	RARA Amp	NRG2 mRNA	NRG2 Amp	Groups	Lapatinib IC50 (μM)
NCIN87	13.68068	ERBB2-H	7.579796	RARA-H	4.240898	NRG2-H	Group 4	0.06610655
AZ521	9.076744	ERBB2-H	5.968132	RARA-H	4.311362	NRG2-H	Group 4	1.659918428
NUGC4	10.21848	ERBB2-H	5.721415	RARA-L	3.852881	NRG2-H	Group 3	0.171543315
MKN7	12.00612	ERBB2-H	6.058238	RARA-H	3.661981	NRG2-L	Group 3	8
RERFGC1B	9.667308	ERBB2-H	5.777195	RARA-L	3.974191	NRG2-H	Group 3	8
NUGC3	8.006484	ERBB2-L	6.300127	RARA-H	3.986192	NRG2-H	Group 3	2.410752535
SNU16	8.917765	ERBB2-H	6.59063	RARA-H	3.545393	NRG2-L	Group 3	6.697770596
FU97	6.999068	ERBB2-L	6.050557	RARA-H	3.820554	NRG2-H	Group 3	8
KE39	9.27483	ERBB2-H	5.956177	RARA-L	3.61809	NRG2-L	Group 2	4.056060314
GCIY	8.439433	ERBB2-L	6.460522	RARA-H	3.708975	NRG2-L	Group 2	7.255415916
MKN74	8.119466	ERBB2-L	6.73371	RARA-H	3.257784	NRG2-L	Group 2	4.689732791
IM95	9.108876	ERBB2-H	5.806562	RARA-L	3.74757	NRG2-L	Group 2	8
HS746T	7.43275	ERBB2-L	5.676293	RARA-L	4.431395	NRG2-H	Group 2	8
SNU1	8.776752	ERBB2-L	5.182644	RARA-L	5.151627	NRG2-H	Group 2	8
OCUM1	9.006604	ERBB2-L	5.674769	RARA-L	3.74211	NRG2-L	Group 1	8
SH10TC	7.577364	ERBB2-L	5.941876	RARA-L	3.724585	NRG2-L	Group 1	8
MKN45	8.476658	ERBB2-L	5.247703	RARA-L	3.581405	NRG2-L	Group 1	8

### ERBB2+ GC Strongly Associates With CIN Subtype of TCGA and Intestinal-Type of Lauren Classification

**FIGURE 5:**
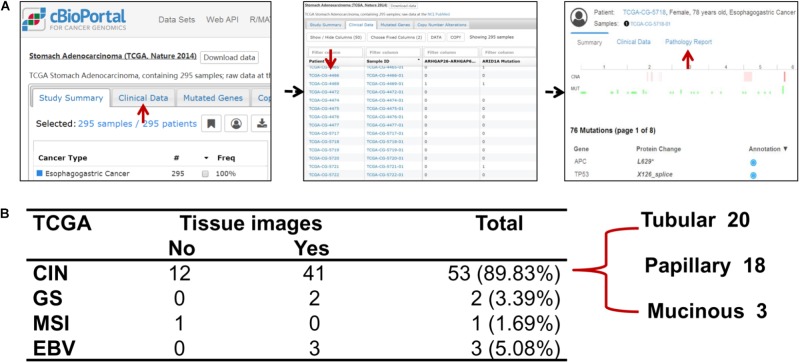
The relationship of molecular classification and clinicopathological types of ERBB2+ GC. **(A)** The flowchart of extracting clinicopathological information of TCGA cohort. **(B)** The molecular classification features of ERBB2+ GC and corresponding clinicopathological types to Lauren classification in TCGA cohort. The predominant molecular classification is CIN subtype, which overlaps with intestinal-type GC of Lauren classification.

The clinical details and histology of TCGA cohort were recorded in cBioPortal database^9^, which provided opportunity to analyze correlation between genomic information and clinicopathological characteristics. For 59 cases of ERBB2+ GC, the pathology reports and slide images of 46 cases were uploaded in database (**Figure 5A**). Fifty-three cases out of 59 ERBB2+ GC (89.83%) belong to chromosome instability (CIN) molecular subtype, and the others include three Epstein-Bar Virus-related (EBV) subtype, two genome stable (GS) subtype, and one microsatellite instability (MSI) subtype (**Figure 5B**). Based on their pathological reports of ERBB2+ GC, all of those are intestinal-type GC in traditional Lauren classification, such as tubular adenocarcinoma, papillary adenocarcinoma, and a few mucinous adenocarcinomas.

### Validating Study of Lapatinib and ATRA Drugs on Different GC Cell Lines

**FIGURE 6:**
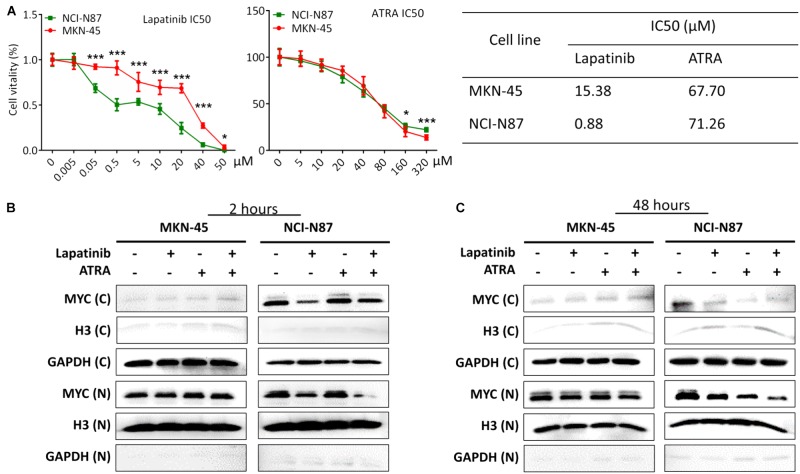
The validating study on NCI-N87 and MKN-45 GC cell lines based on drug Lapatinib and ATRA treatment. **(A)** Analyzing IC50 of Lapatinib and ATRA on MKN-45 and NCI-N87 cell lines. NCI-N87 is a cell line with ERBB2 and RARA amplification, while MKN-45 is a cell line without ERBB2 and RARA amplification. **(B)** Examination of MYC protein levels on cytoplasm and nucleus after incubation by Lapatinib (0.05 μM) or ATRA (50 μM) or both drugs for 2 h. **(C)** Examination of MYC protein levels on cytoplasm and nucleus after incubation by Lapatinib (0.05 μM) or ATRA (50 μM) or both drugs for 48 h. “C” represents cytoplasmic protein. “N” represents nuclear proteins. ^∗^*P* < 0.05; ^∗∗∗^*P* < 0.001.

Based on data analysis of multiple GC cell lines (**Table 2**), cell line NCI-N87 (ERBB2-H/RARA-H) and MKN-45 (ERBB2-L/RARA-L) were selected for further validating experiments. The IC50s of Lapatinib (targeted to ERBB2) and ATRA (targeted to RARA) were assayed by CCK8 method on NCI-N87 and MKN-45 cell lines. As showed in **Figure 6A**, cell line NCI-N87 was more sensitive than cell line MKN-45 to drug Lapatinib (IC50 0.88μM vs. 15.38 μM, *P* < 0.001), while there was no significant difference of IC50s of ATRA in both GC cell lines (71.26μM vs. 67.70 μM, *P* = 0.281). By further CI analysis, synergistic effect of ATRA (25 μM) and Lapatinib (between 0.005 and 0.5 μM) was observed on NCI-N87 cell line, but not in MKN-45 cell line (**Table 3**).To verify the effect on Myc signaling pathway, the cytoplasmic protein and nuclear protein were extracted separately, and examined expressing levels after incubating Lapatinib, ATRA or both. As results, after 2 h incubation of Lapatinib (0.05 μM), ATRA (25 μM), or both on NCI-N87 and MKN-45 cells, obvious down-regulation of MYC protein levels of cytoplasm and nucleus was found in Lapatinib treated NCI-N87 cells, but bot ATRA did. Importantly, incubation of Lapatinib and ATRA simultaneously further reduced nuclear protein level of MYC in NCI-N87 cells (**Figure 6B**, right). The similar effects were also observed on NCI-N87 cells after 48 h incubation by Lapatinib and ATRA, or both (**Figure 6C**, right). However, these effects were not observed in MKN-45 cells (**Figures 6B,C**, left).

**Table 3:** Analysis of combination index for Lapatinib and ATRA (25 μM).

Lapatinib (μM)	NCI-N78		MKN-45	
	IR	CI	IR	CI
0.005	0.31	0.70	0.08	3.28
0.05	0.64	0.24	0.20	1.35
0.5	0.77	0.41	0.26	2.18
5	0.77	3.16	0.43	8.28

*CI, combination index; IR, inhibition rate.*

## Discussion

ERBB2 plays a critical role in carcinogenesis and cancer progression and has become a targeted therapeutic molecule in breast cancer and GC. Exploring ERBB2 related pathways and mechanisms of resistance will help oncologist to overcome drug resistance and enhance therapeutic effect of ERBB2-targeted therapy (Shi et al., 2016). Clinically, not all ERBB2+ GC respond well to ERBB2-targeted treatment due to innate resistance or acquired resistance (Kim et al., 2014; Piro et al., 2016). Therefore, exploring resistant mechanism of ERBB2+ GC is desirable. Han et al found that SRC inhibitor combined with trastuzumab can synergistically inhibit proliferation of ERBB2+ GC *in vitro* (Han et al., 2014).

Two decades passed since trastuzumab was used for treatment of ERBB2+ breast cancer (Goldenberg, 1999; Shak, 1999; Sakamoto and Mitsuyama, 2000). Some ERBB family inhibitors, such as Lapatinib and Afatinib are also developed for treatment of ERBB2+ breast cancer or GC (Geuna et al., 2012; Huang and Rizzo, 2012; Lin et al., 2012; Schuler et al., 2012; Hecht et al., 2016). Large amount of clinical information as well as their corresponding genomic changes has been submitted to open databases. Cross-database analysis is a challenging work, but it helps researchers to get an overview of interested problems. In this study, we performed a cross-database data mining for factors involved in sensitivity or resistance to ERBB2-targeted therapy in ERBB2+ GC. We found that both ERBB2 and RARA are involved in Myc signaling pathway. Myc is the important hub gene of both ERBB2 and RARA. By data mining, a total of 47 genes were outlined as potential targets of treatment for ERBB2+ GC. We noticed that RARA, THRA, CACNB1, and TOP2A are targets of FDA-approved drugs. For instance, CACNB1 is a calcium channel protein and the inhibitor amlodipine is one of the most popular medications for treatment of high blood pressure (Gregg et al., 1993; Nussberger et al., 2008). TOP2A is the target of etoposide, which has been used in cancer treatment (Slater et al., 2001; Bartlett et al., 2015). RARA, which participates in regulation of cell development, differentiation, apoptosis and granulopoiesis. ATRA is a RARA agonist, which revealed clinical efficacy in leukemia treatment (Degos and Wang, 2001). THRA is thyroid hormone receptor alpha, whose ligand tiratricol was used in treatment of hypothyroidism (Yazdanparast et al., 2006; Tylki-Szymanska et al., 2015). GSEA is a powerful tool to find biological pathways for associated genes. By GSEA method, all amplified genes ERBB2, RARA, THRA, CACNB1, and TOP2A are identified to be related to Myc signaling pathway. Myc plays important roles in carcinogenesis and cancer progression (Sun et al., 2011; Stine et al., 2015). In gastric carcinogenesis, pathogen infection of *H. pylori* and EBV could activate Myc pathway (Calcagno et al., 2008). Our cross-database analysis suggests that inhibitor of TOP2A and agonist of RARA, THRA, or CACNB1 may have synergistic efficacy in ERBB2-targeted therapy for ERBB2+ GC. Li and coworkers found that ERBB2 activated downstream Myc and promoted cells proliferation by phosphorylating AKT1 and Erk1/2 in cervical cancer (Li et al., 2018). RARA could interact with Myc and regulate RARA-dependent genes expression in leukemia cells (Uribesalgo et al., 2012).

In this study, three potential ligands (SH3BGRL3, SH3BGRL, and NGR2) of ERBB2 have been proposed. The latter has been reported as a ligand for ERBB3/ERBB4 (Chang et al., 1997; Schulze et al., 2005; Chiang et al., 2015). Slattery and coworkers found that NRG2 as a growth factor was involved in progression of breast cancer (Slattery et al., 2013). Sun and colleagues found that both NRG1 and NRG2 functioned as ligands of ERBB family and involved in progression of non-small cell lung cancer (Sun et al., 2009). Hitherto, the relationship of NRG2 and ERBB2 in GC is not clear yet. Our analysis disclosed that overexpression of NRG2 is significantly associated with poor prognosis of ERBB2+ GC. Some reports suggested that NRG2 can promote phosphorylation of ERBB2, ERBB3, and ERBB4 directly or indirectly (Sliwkowski et al., 1994; Chang et al., 1997; Mill et al., 2011). Therefore, NRG2 as a potential ligand of ERBB2 is worth further explored. Regarding to SH3BGRL3 and SH3BGRL, there is no any report on the relationship of these genes with ERBB2. In the present study, the simultaneous overexpression of ERBB2, NRG2, and RARA were correlated with increased sensitivity to Lapatinib. We speculate that ERBB2, NRG2, and RARA might act together as a functional network.

It has been reported that there are some histopathological features of ERBB2+ GC (Park et al., 2016). ERBB2 positivity is more often observed in intestinal-type GC (He et al., 2013; Irkkan et al., 2017). Up-to-date, there is no correlation analysis on both levels of genomics and clinical histology for ERBB2+ GC. TCGA database provides a good resource for comparative analysis on both levels because it stores perfect genomics and histological information for GC samples. In 59 cases of ERBB2+ GC, 46 cases were accompanied by whole slide tissue images. ERBB2+ GC revealed characteristic histopathology. All 46 cases were classified into intestinal-type GC using Lauren classification, mainly including tubular adenocarcinoma, papillary adenocarcinoma, and a few mucinous adenocarcinomas. The majority (near 90%) of ERBB2+ GC was classified into CIN subtype on genomic level using TCGA molecular classification (Bass et al., 2014). These findings provide an important clue for clinical selection of ERBB2-targeted therapy.

In our validating study, we confirmed that GC cell line with amplified ERBB2 and RARA (NCI-N87) was sensitive to Lapatinib relative to GC cell without amplified ERBB2 and RARA (MKN-45). The sensitivity to ATRA on both cell lines did not show significant difference, which may attribute to some alternative pathways for ATRA-mediated apoptosis (Patrad et al., 2018). We found that Lapatinib and ATRA could synergistically inhibit cell growth in NCI-N87 cell, but not in MKN-45 cell. These biological effects were involved in interfering Myc signaling pathway. The similar effect was reported for breast cancer (Paroni et al., 2012).

**FIGURE 7:**
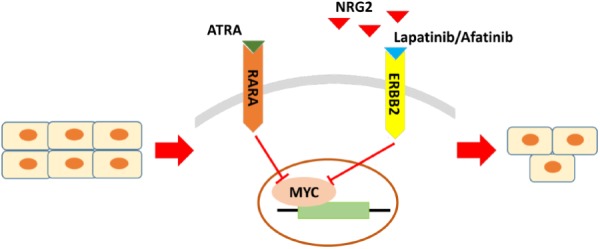
The crucial molecules and regulatory pathways of ERBB2+ GC. The main regulatory pathway is NRG2-ERBB2-MYC, which is cross-interfaced with RARA-MYC pathway. For ERBB2+ GC, beyond ERBB2 itself, RARA and NRG2 are potential drug sensitive biomarkers too for predicting response to ERBB2-targeted therapy. Combination of NRG2 antagonist or RARA agonist with ERBB2-targeted drug may be effective regimens for ERBB2+ GC.

In summary, by cross-database analysis, GC with increased expression of ERBB2, RARA, and NRG2 simultaneously should be taken as sensitive category to Lapatinib therapy. The current study proposed that NRG2-ERBB2-MYC is cross-interfaced with RARA-MYC pathway. Intervention of both routes will regulate hub gene Myc, and then inhibit cell growth for ERBB2+ GC (**Figure 7**). Moreover, combination of RARA agonist ATRA with ERBB2-targeted drug disclosed synergistic anticancer effect for ERBB2+ GC *in vitro*.

## Author Contributions

YY, XH, and ZZ were involved in concept and design. ZX, JW, and JZ acquired the data and performed data mining. ZX, JJ, and RY performed the experiments. WC and YY supported the analysis. All authors wrote, reviewed, and revised the manuscript.

## Conflict of Interest Statement

The authors declare that the research was conducted in the absence of any commercial or financial relationships that could be construed as a potential conflict of interest.

## Footnotes

1 https://cancergenome.nih.gov/

2 http://www.cbioportal.org/

3 https://www.ncbi.nlm.nih.gov/geo/

4 https://www.intomics.com/inbio/map/#home

5 http://www.broadinstitute.org/ccle

6 http://portals.broadinstitute.org/ctrp.v2.1/

7 http://www.genome.jp/kegg/pathway.html

8 https://www.proteinatlas.org/

9 http://www.cbioportal.org/study?id=stad_tcga_pub#clinical
